# Bouncing and spinning of amorphous Lennard-Jones nanoparticles under oblique collisions

**DOI:** 10.1038/s41598-022-14754-1

**Published:** 2022-06-23

**Authors:** Maureen L. Nietiadi, Herbert M. Urbassek

**Affiliations:** Physics Department and Research Center OPTIMAS, University Kaiserslautern, Erwin-Schrödinger-Straße, Kaiserslautern, D-67663 Germany

**Keywords:** Astronomy and planetary science, Physics

## Abstract

Collisions of Lennard-Jones nanoparticles (NPs) may be used to study the generic collision behavior of NPs. We study the collision dynamics of amorphous NPs for oblique collisions using molecular dynamics simulation as a function of collision velocity and impact parameter. In order to allow for NP bouncing, the attraction between atoms originating from differing NPs is reduced. For near-central collisions, a finite region of velocities – a ‘bouncing window’ – exists where the 2 NPs bounce from each other. At smaller velocities, energy dissipation and – at larger velocities – also NP deformation do not allow the NPs to surpass the attractive forces such that they stick to each other. Oblique collisions of non-rotating NPs convert angular momentum into NP spin. For low velocities, the NP spin is well described by assuming the NPs to come momentarily to a complete stop at the contact point (‘grip’), such that orbital and spin angular momentum share the pre-collision angular momentum in a ratio of 5:2. The normal coefficient of restitution increases with impact parameter for small velocities, but changes sign for larger velocities where the 2 NPs do not repel but their motion direction persists. The tangential coefficient of restitution is fixed in the ‘grip’ regime to a value of 5/7, but increases towards 1 for high-velocity collisions at not too small impact parameters, where the 2 NPs slide along each other.

## Introduction

Collisions of particles (also termed ‘grains’) play an important role in several branches of science. In environmental physics, the size distribution of aerosols is influenced by their collision dynamics^1,2^. In chemical engineering, powder technology is based on the manipulation of grains; again, the collisional interaction of grains may influence their size and properties^3^. In protoplanetary disks – where particles are conventionally termed dust – agglomeration of dust is the first step leading eventually to the building of asteroids and even planets^4–7^. Also in other situations of astrophysical interest, dust collisions are relevant such as in debris disks of developed planetary systems^8,9^, in planetary rings^10^, and in the dust tails of comets^11,12^.

While collision experiments are readily performed on particles of mm- and even $\mu$m-size^13,14^, collisions of nanoparticles (NPs) are most easily studied using theoretical tools such as analytical models^15–17^ or simulation^18^. In the lowest size regime, molecular dynamics (MD) simulation has shown as a promising venue to deliver information on collisions of NPs with sizes of a few ten nm. Here, NPs of a variety of materials have been studied, among them silica^19^, water ice^20^, and also core-shell structures^21,22^. Interesting results that have been achieved include the dependence of the bouncing velocity – that is the velocity discriminating the low-velocity sticking from the high-velocity bouncing outcomes of a collision – on material characteristics and NP size.

As a rule, these studies have been performed for central collisions^23^, as these are essentially one-dimensional and reduce both the parameter space to investigate and the number of observables to analyze. However, the consideration of oblique collisions opens up new questions such as the collisional excitation of NP rotation or the influence of the impact parameter on the bouncing velocity.

In the present paper, we use MD simulation to study oblique collisions in a generic system: NPs that are composed of Lennard-Jones (LJ) material. LJ has served for several decades as a prototypical material^24–26^ since its atoms interact via a simple pair potential and the results obtained obey simple scaling rules that often allow their transfer to other systems of interest^18,27–35^. It is thus hoped that the present results can provide a general insight into the deviations that occur between oblique and central NP collisions. We shall study the collision modes – from sticking to bouncing – the (normal and tangential) coefficients of restitution, excitation of NP spin, the change of the bouncing velocity with impact parameter, and NP deflection under the collision.

## Method

Atoms interact via the standard Lennard-Jones (LJ) potential

1 $$\begin{aligned} V(r) = 4 \epsilon \left[ \left( \frac{\sigma }{r} \right) ^{12} - \left( \frac{\sigma }{r} \right) ^{6} \right] \end{aligned}$$

with length parameter $\sigma$ and energy parameter $\epsilon$; the potential is cut off at $r_c=5\sigma$ such that $V(r)=0$ for $r>r_c$. In the following, we will use LJ units: The unit of time is $\tau =\sigma \sqrt{m/\epsilon }$, where *m* is the atom mass; lengths are measured in units of $\sigma$, and the unit of velocity is $\sqrt{\epsilon /m}$. As an example, for the case of Ar clusters, it is $\epsilon = 10.32$ meV and $\sigma = 3.41$ Å^36–38^; hence the units of time and velocity correspond to 2.16 ps and 158 m/s, respectively.

Amorphous LJ NPs are obtained by rapidly quenching from the melt^39,40^. In detail, a cubic box of box length 35.4 is filled with liquid LJ material at a temperature of $T=2.5$ and slowly quenched under periodic boundary conditions with a quench rate of 0.0018 in LJ units until the temperature is close to 0. A sphere of radius $R=88.23$ containing 3,131,097 atoms is cut out from a sufficiently large array of the periodic images of this box and then relaxed in an NVE ensemble for a LJ time of 25 in order to obtain relaxed surfaces.

**Figure 1:**
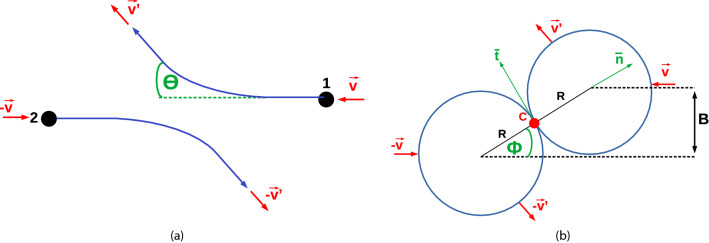
(**a**) Sketch of the collision of NP1 and NP2 in the center-of-mass frame. The initial collision velocity of NP1 is $\varvec{v}$, its post-collision velocity is $\varvec{v}'$; they make an angle $\theta$. Because of symmetry, NP2 has – apart from the sign – the same pre- and post-collision velocities and deflection angle as NP1. (**b**) Sketch of the collision kinematics at the moment where the two NPs touch at point C. C is also the – unmoving – center of mass of the system. The blue circles denote the NPs, *R* is the NP radius, *B* the impact parameter, and the angle $\phi$ is given by Eq. (3).

For the collision simulation, one NP is duplicated and put outside of the cut-off radius of the first NP; if several simulations are performed with identical velocity and impact parameter, one of the NPs is rotated by a random angle before starting the simulation. The two NPs are given an initial velocity of $\varvec{v}$ and $-\varvec{v}$ with equal magnitude but opposite direction, see Fig. 1a. Thus, the collision is simulated in the center-of-mass- frame; the relative velocity of the 2 NPs is 2*v*. The impact parameter *B* is given by the initial distance of the 2 NPs in the direction perpendicular to $\varvec{v}$, see Fig. 1b. We define the relative impact parameter as

2 $$\begin{aligned} b = \frac{B}{2R} . \end{aligned}$$

Central collisions are characterized by $b=0$ and glancing collisions by $b=1$.

We found that the amorphous NPs are sticking for central impacts for all velocities; this is in contrast to crystalline LJ NPs^30,35^. In order to improve the tendency for bouncing, we modified the attraction between atoms of different NPs by reducing the LJ parameter $\epsilon$ in Eq. (1) to a parameter $\epsilon _{12}$, while the interaction between atoms of the same NP is unaltered. Such a changed interparticle interaction might for instance be caused by contaminants adsorbed on the NP surfaces. With this modification, we found bouncing if $\epsilon _{12}=0.2$ and fixed this value in the present study. No systematic research into the dependence of our results on the choice of $\epsilon _{12}$ was performed. However, in the limit of vanishing interparticle attraction, all collisions will be bouncing, such that a relevant collision channel entirely disappears. We note that also in previous simulation studies of LJ NP collisions, a reduction of the interparticle attraction was introduced^29,32–34^.

The simulations are run until the 2 NPs separated from each other at least by the cut-off radius of the potential. If the 2 NPs stick after the collision, the termination of the simulation is to some degree arbitrary; for determining the lower bouncing velocity, $v_b$, we waited a factor of 5–10 longer than the nearest bouncing case to see if the 2 NPs are really stuck together. In the case of fragmentation, which occurred for high velocities, we also stopped the simulation before fragmentation was complete, since the study of fragmentation processes is not in the center of this work.

Note that because of the symmetry of the collision scenario, it is sufficient to consider the motion of one NP. The position, velocity and rotational velocity (spin angular momentum) of the second particle can be obtained from the conservation of momentum and angular momentum.

The total angular momentum is calculated by summing $m\varvec{r}_i \times \varvec{v}_i$ over all atoms *i* (mass *m*) with respect to the fixed center of mass, C, of the system. Analogously, the spin angular momentum of NP1 is calculated from the analogous expression by summing over all atoms that belong to NP1 and referring positions and velocities to the (moving) center of mass of NP1; analogous for NP2. The orbital angular momentum is given by the analogous expression, summing over the center-of-mass positions and velocities of NP1 and 2.

The molecular dynamics simulations are performed with the LAMMPS code^41^. Atomistic snapshots are generated with OVITO^42^.

**Figure 2:**
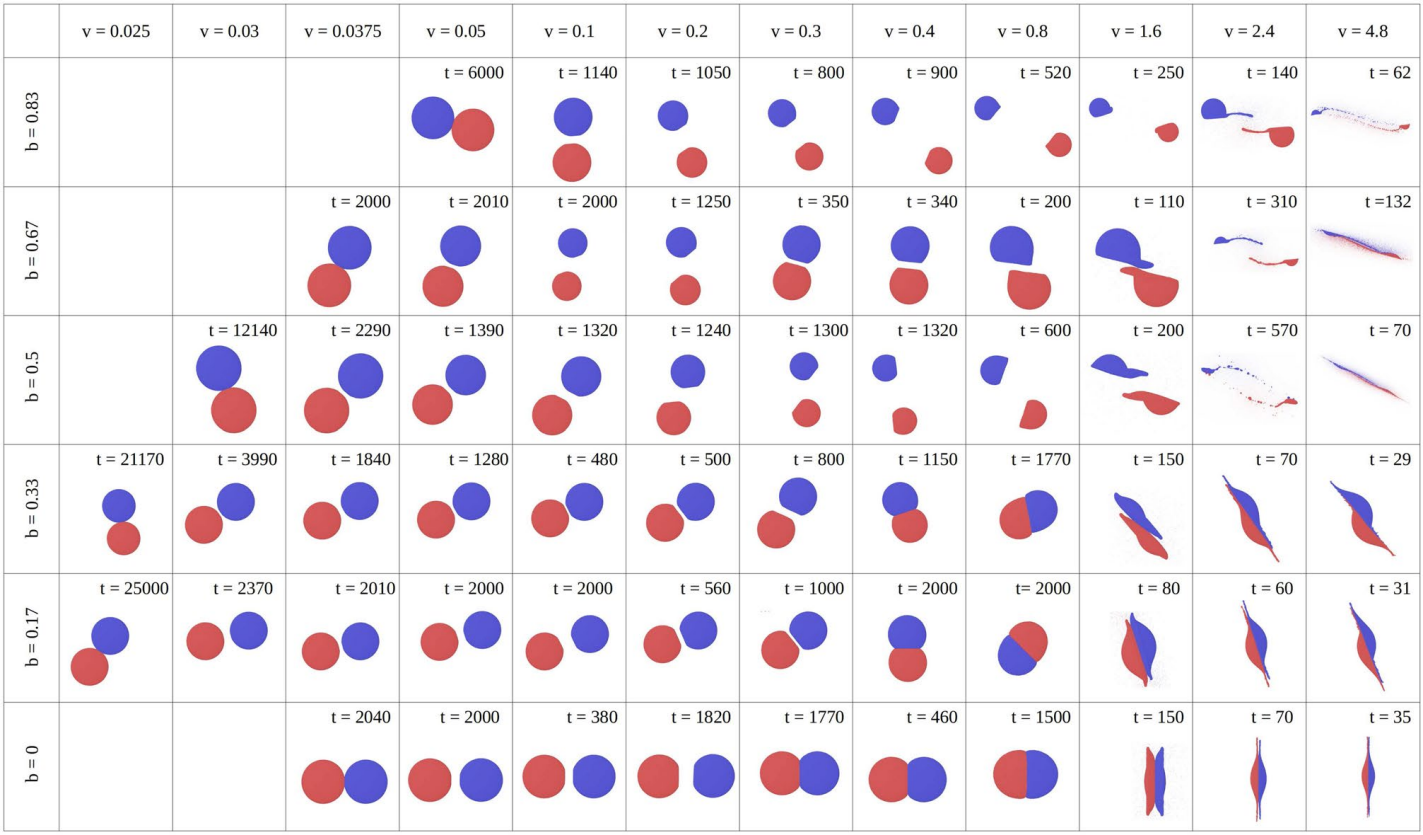
Overview over the post-collision configurations of 2 NPs colliding with velocity *v* and impact parameter *b*. Colors denote original association of atoms to NP 1 and 2. Snapshots were taken at the times *t* indicated, when the collision outcome – bouncing or sticking – can be determined from the separation between the two NPs.

**Figure 3:**
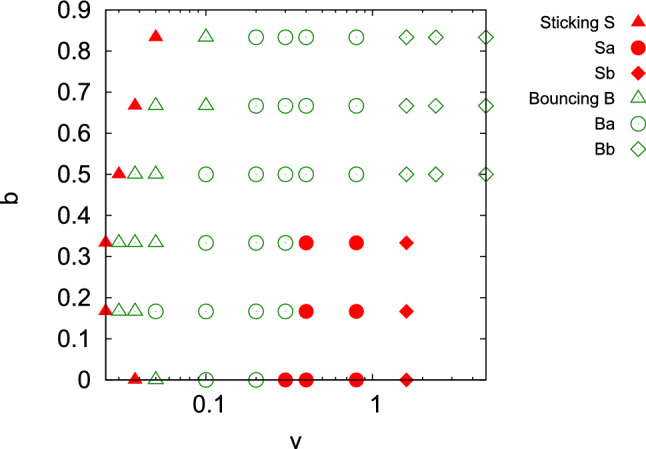
Classification of NP collisions into sticking and bouncing in dependence of collision velocity *v* and impact parameter *b*. Filled symbols: sticking collisions (S), with Sa: sticking and flattening, and Sb: sticking and strong deformation (shearing, fragmentation). Open symbols: bouncing collisions (B), with Ba bouncing and flattening, and Bb: bouncing and strong deformation (sliding, fragmentation). Note the logarithmic abscissa scale.

## Results

### Overview over collision modes – Classification of collisions

The outcomes of NP collisions can quite generally be classified into ‘sticking’ and ‘bouncing’ collisions depending on whether the NPs separate again after impact or not; at high velocities, in addition NP fragmentation will set in.

Figure 2 gives an overview of these outcomes for several velocities and impact parameters. The snapshots were taken at the time when the simulation was terminated, cf. Sect. “Method”. Quite generally, we observe sticking at low velocities, followed by bouncing at higher velocities. For small impact parameters $b\le 0.33$, a second sticking regime is encountered at even higher velocities, which is caused by strong energy dissipation during the collision. The bounced NPs show deformation after the collision. At the smallest velocities, the plastic deformation is concentrated at the collision zone and may be termed ‘flattening’. At the largest velocities, the deformation spreads out over the entire NP and even fragmentation sets in. Figure 3 shows how the sticking and bouncing collisions depend on the velocity and impact parameter. In this plot, we tentatively subdivided the sticking (S) and bouncing (B) regimes further by denoting a small letter indicating flattening (a) and strong deformation (b); the exact boundary between flattening and strong deformation is somewhat subjective. For negligible NP deformation, the pure symbols S and B are used. The low-velocity boundary between sticking and bouncing collisions will analyzed in detail in Sect. “Bouncing velocity”.

For non-central collisions in the sticking regime, the merged NPs spin around each other. In such a case, the snapshots of Fig. 2 give only an instantaneous look on the rotating system. For non-central bouncing collisions, the 2 NPs have escaped each other at the time when the snapshots were taken; each NP will spin around its center. These rotational motions will be further analyzed in Sect. “Angular momentum”.

A typical feature seen in Fig. 2 is the compression (‘flattening’) of NPs in their contact area. This compression starts already at quite small velocities and has not been observed for crystalline LJ NPs^35^. We therefore believe that it is caused by the soft surface structure of amorphous NPs. A characteristic feature of larger-impact-parameter collisions is a ‘shaving’ (or ‘sliding’^18^) process where the contact area of NPs is not only flattened but NPs leave the contact zone again because lateral friction does not allow them to reduce their velocity sufficiently for sticking, see e.g. $b=0.67$, $v=0.8$. At higher velocities, material is moved out sideways from the collision zone by friction at the contact zone ($b=0.67$, $v=1.6$); this removed material forms long filaments ($b=0.67$, $v=2.4$) until the two NPs become strongly fragmented at even higher velocities.

For smaller impact parameters, bouncing NPs show mild deformations at the contact zone. In the higher-velocity regime of sticking, the NPs weld at their contact zone ($b=0.33$, $v=0.8$) but start sliding along it for higher velocities ($b=0.33$, $v=1.6$). This change in transverse-momentum accommodation will be discussed in more quantitative detail in Sect. “Normal and tangential restitution coefficients”. At even higher velocities, the NPs are strongly deformed to pancake-like structures, which, however, continue to adhere to each other ($b \le 0.33$, $v=4.8$).

A similar map, albeit for smaller NPs, $R=13.5$, including similar collision outcomes has been obtained previously^18^. The features of low-velocity sticking, increasing fragmentation at large velocities and ‘sliding’ collisions at larger impact parameters are common for both NP sizes. As novel features we find a bouncing regime as well as a second (higher-velocity) sticking regime at low impact parameters; also, the shape of the low-velocity sticking-bouncing threshold becomes more complex as will be discussed in Sect. “Bouncing velocity”.

### Normal and tangential restitution coefficients

The velocity changes caused by the collision are conveniently described by the restitution coefficients. We consider the collision in the center-of-mass frame of the 2 NPs, in which the center of mass of the system, C, is fixed. C is also the point at which the 2 NPs would touch first if they moved on straight trajectories without interaction, see Fig. 1b. The angle $\phi$ between the line connecting the centers of the 2 NPs and the initial velocity $\varvec{v}$ is given by

3 $$\begin{aligned} \sin \phi = \frac{B}{2R} = b . \end{aligned}$$

We set up a coordinate system in C with axis $\varvec{n}$ pointing towards the center of sphere 1, and axis $\varvec{t}$ tangential to the 2 NPs, see Fig. 1b. $\varvec{n}$ and $\varvec{t}$ are unit vectors. Then the initial velocity of NP1 can be expressed as

4 $$\begin{aligned} \varvec{v} = v_n \varvec{n} + v_t \varvec{t} , \end{aligned}$$

with the normal component $v_n = -v \cos \phi$ and the tangential component $v_t = v \sin \phi$.

After the collision, the post-collision velocity of NP1, $\varvec{v}'$, can be similarly expressed as

5 $$\begin{aligned} \varvec{v}' = v'_n \varvec{n} + v'_t \varvec{t} . \end{aligned}$$

The tangential and normal components of $\varvec{v}'$ can be expressed as

6 $$\begin{aligned} v'_n = e_n v_n, \quad v'_t = e_t v_t , \end{aligned}$$

where $e_n$ ($e_t$) are the normal (tangential) coefficients of restitution. Note that in some literature^43–46^, different definitions of these quantities are given – $e_n$ may have the opposite sign, and $e_t$ may refer to the instantaneous speed of atoms at point C including (pre- and post-collisional) NP spin. For our purposes, the definitions in Eq. (5) appear to be most appropriate, as they allow immediately to calculate $\varvec{v}'$ from $\varvec{v}$ once $e_n$ and $e_t$ are known. These coefficients of restitution describe the *persistence* of motion after the collision: Values of 1 indicate that the NPs move on unperturbed by the collision; this will occur at grazing collisions. A central impact with $e_n=-1$ denotes a totally elastic reflection; collisions with $e_n=0$ and $e_t=0$ denote a sticking collision.

**Figure 4:**
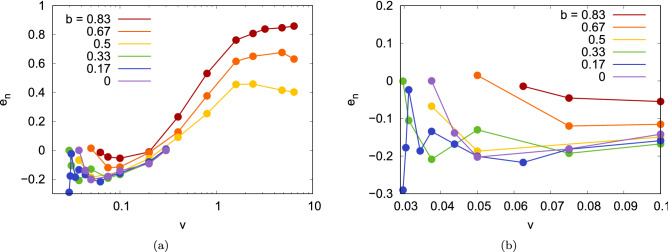
(**a**) Normal restitution coefficient, $e_n$, as a function of collision velocity *v* for several impact parameters *b*. (**b**) zooms into the low-velocity region. Data are only shown for bouncing collision. Note the logarithmic abscissa scale in subpanel (**a**).

Figure 4a displays the normal coefficient of restitution as calculated from our simulations; Fig. 4b zooms into the low-velocity region. For central impacts, we find that the normal coefficient of restitution assumes small negative values, $-0.2< e_n < 0$, in the bouncing regime, $0.03 \le v \le 0.4$. $e_n$ is negative in our definition Eq. (6) since the NP velocity is reversed during bouncing. The smallness of $e_n$ indicates that the collision is strongly inelastic and a considerable part of the collision energy is dissipated. In this velocity regime, $e_n$ is negative also for larger impact parameters. Quantitatively $e_n$ does not change much with *b* up to $b \le 0.5$; only for higher impact parameters, its values change and approach 0.

For the large impact parameters which show bouncing also at high velocities ($b \ge 0.5$), $e_n$ assumes positive values for $v \ge 0.4$. Since these are near-glancing collisions, even the normal component of $\varvec{v}$ persists after the collision; for $b=0.83$ and the highest velocities, $e_n$ approaches 1 as in an unperturbed trajectory.

**Figure 5:**
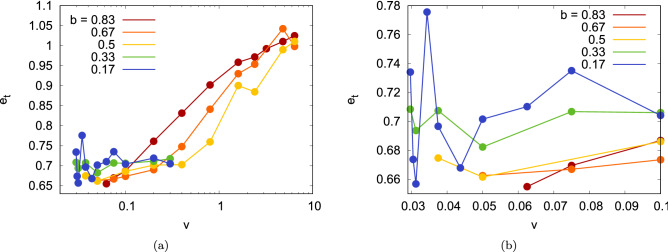
(**a**) Tangential restitution coefficient, $e_t$, as a function of collision velocity *v* for several impact parameters *b*. (b) zooms into the low-velocity region. Data are only shown for bouncing collision. Note the logarithmic abscissa scale in subpanel (**a**).

The dependence of the tangential coefficient of restitution on velocity and impact parameter is displayed in Fig. 5a,b zooms into the low-velocity region. Interestingly, at small velocities, it is $e_t \sim 0.7$ for all impact parameters. This behavior will be discussed in detail in Sect. “Angular momentum”; there it will be shown that when assuming that the 2 NPs come to a complete stop at the collision point, the conservation of total angular momentum predicts $e_t=5/7$, Eq. (12), in fair agreement with our simulation results for velocities $v \le 0.4$ for the small impact parameters ($b \le 0.5$). For larger impact parameters, and for $b=0.5$ for larger velocities, $e_t$ increases towards 1, as expected for a grazing collision, in which the NPs change their velocity only negligibly.

Large fluctuations show up in the coefficients of restitutions for small velocities. As shown in detail below (Figs. 10 and 11 and their discussion in Sect. “Bouncing velocity”) the details of the surface structure of amorphous NPs determines the outcome of a collision in the vicinity of the bouncing velocity. Hence the exact values of the bouncing velocity – and similarly the coefficients of restitution and the spin excitation – depend on the relative orientations of the two NPs and show hence a large sensitivity on the collision velocity and impact orientation.

**Figure 6:**
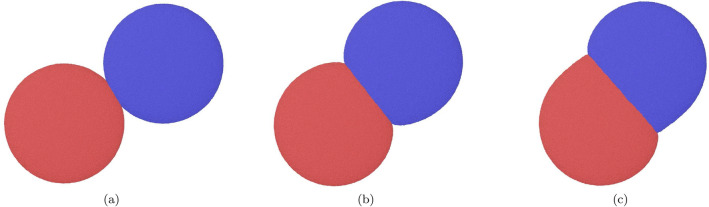
Snapshots showing the collision with impact parameter $b=0.5$ around the moment of closest approach for collision velocity (**a**) $v=0.05$, (**b**) 0.4, and (**c**) 0.8.

### Angular momentum

In the center-of-mass frame, the collision is symmetric against a permutation of NP1 and NP2: $\varvec{v}_1 = - \varvec{v}_2$ and $\varvec{R}_1 = - \varvec{R}_2$, where $\varvec{v}_i$ and $\varvec{R}_i$ denote the instantaneous velocities and positions of the center of mass of NP *i*. It is therefore sufficient to focus on the angular momentum of NP1 in the following, since the angular momentum of NP2 is identical. Since the NPs are initially non-rotating, the initial angular momentum is entirely composed of the orbital momentum around the center of mass, C, and has only a component perpendicular to the plane; we will denote this component by *L*. From Fig. 1b, we see that

7 $$\begin{aligned} L = \frac{1}{2} MvB = MvR b = MR v_t . \end{aligned}$$

This value is conserved throughout the entire collision, but after the collision, *L* is composed of both a spin and an orbital component,

8 $$\begin{aligned} L' = L = L'_\mathrm{orb} + L'_\mathrm{spin}, \end{aligned}$$

where $L'$ denotes the post-collision angular momentum.

Since the total angular momentum is conserved, it is sufficient to discuss one component of $L'$; we focus on the spin. The behavior of $L'_\mathrm{spin}$ is analyzed most readily at low velocities. Here, an inspection of the NP trajectories shows that at the moment of contact, lateral friction between the two colliding NPs perfectly adjusts their tangential velocities, see Fig. 6a. This means that the tangential velocity of atoms of NP1 at point C, $v_C = v'_{t} - \omega ' R$, must be equal to the tangential velocity of atoms of NP2 at point C; because of symmetry this means, it must be zero,

9 $$\begin{aligned} v_C = v'_t - \omega ' R =0. \end{aligned}$$

Here $\omega '$ denotes the post-collision angular velocity of NP1; it is identical to that of NP2. Eq. (9) relates $\omega '$ to $v'_t$, such that

10 $$\begin{aligned} \omega ' = \frac{v'_t}{R} . \end{aligned}$$

A second relation is obtained from angular-momentum conservation, Eq. (8), which may be written as

11 $$\begin{aligned} MR v_t = MR v'_t + \frac{2}{5} MR^2 \omega ' \end{aligned}$$

with the NP mass *M* and the moment of inertia $2MR^2/5$, since $L'_\mathrm{orb} = MR v'_t$ and $L'_\mathrm{spin} = 2M R^2 \omega ' /5$. Eqs. (9) and (11) thus imply

12 $$\begin{aligned} e_t= \frac{5}{7} . \end{aligned}$$

From Eq. (9) and Eq. (11) we thus obtain the relative angular momenta after collision

13 $$\begin{aligned} \frac{L'_\mathrm{orb}}{L} = e_t = \frac{5}{7} , \quad \frac{L'_\mathrm{spin}}{L} = \frac{2}{7}, \quad \frac{L'_\mathrm{spin}}{L'_\mathrm{orb}} = \frac{2}{5} . \end{aligned}$$

**Figure 7:**
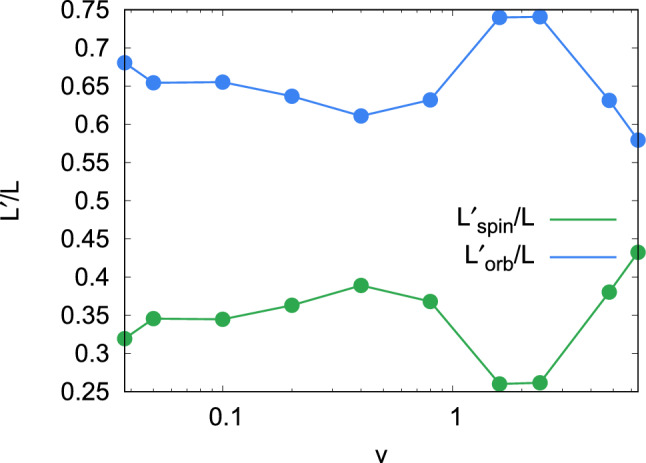
Relative spin and orbital momenta as a function of collision velocity *v* after collision with impact parameter $b=0.5$. Note the logarithmic abscissa scale.

Figure 7 shows the dependence of $L'_\mathrm{orb}$ and $L'_\mathrm{spin}$ on collision velocity for the example of $b=0.5$. We see that this relation, Eq. (13), is well fulfilled for small *v*. We denote the condition of vanishing relative velocities of atoms NP1 and NP2 at point C, Eq. (9), as ‘grip’^45,46^.

**Figure 8:**
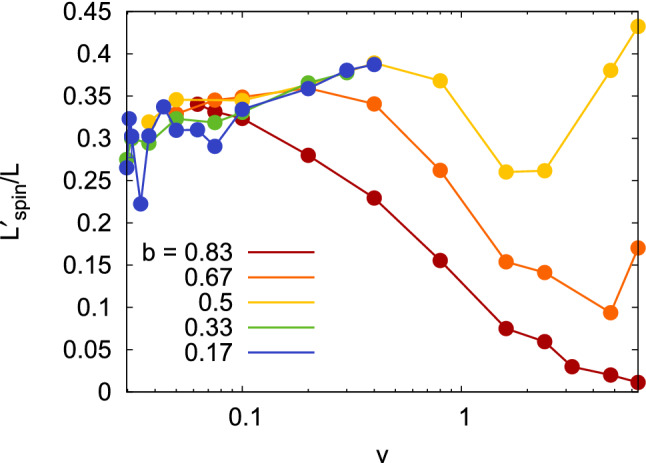
Relative spin momenta as a function of collision velocity *v* after collision for several impact parameters *b*. Data are only shown for bouncing collisions. Note the logarithmic abscissa scale.

The entire evolution of the spin angular momentum with velocity for the impact parameters investigated is shown in Fig. 8; only data for bouncing cases have been included. At low velocities, the relationship $L'_\mathrm{spin} / L = 2/7$, Eq. (13), is well fulfilled.

The increase in $L'_\mathrm{spin} / L$ at small velocities is connected to the increasing compression of the NPs upon close contact. As Fig. 6b shows for the moment of closest approach, with increasing velocity not only the contact area of the 2 NPs broadens, but also distance of the contact area to the center of mass of the NPs shortens from *R* to $R'<R$. As a consequence, the post-collisional angular velocity increases by virtue of Eq. (10) to $\omega ' = 5 v_t /(7R')$ and hence the spin angular momentum increases.

With further increase of *v*, $v>0.5$, spin excitation decreases again. This can be understood by assuming that the ‘grip’ is loosened with increasing velocity, see Fig. 6c: Instead of Eq. (9), we thus postulate $v'_t + \omega ' R =\alpha v_t$ with a positive $\alpha$. An analogous calculation then gives $L'_\mathrm{spin} / L = 2(1 - \alpha )/7$; that is, a loss of grip leads to a decrease in spin excitation, until for $\alpha =1$ the collided NPs have only orbital angular momentum. Fig. 2 shows that this loss of grip occurs in the ‘shaving’-mode collisions in this velocity regime. At even higher velocities, $v>2.4$, strong NP fragmentation makes our analysis inaccurate because more than 2 NPs exist after the collision.

The analysis of our MD simulations thus showed that the grip condition, Eq. (9), holds true for $v \lesssim 0.5$, with the exception of very glancing collisions, $b=0.83$. We do not believe that the range of validity of Eq. (9) could be easily stated a priori; rather, it is only the MD simulations that allowed to establish the validity of the grip condition.

Figure 8 shows a synopsis of the relative spin excitation as a function of velocity and impact parameter. The behavior for $b=0.5$ discussed above, is typical also for the other impact parameters. At low velocities, $L'_\mathrm{spin} / L$ is close to the value of 2/7 for all impact parameters. With increasing velocity, for small impact parameters, it slightly increases, due to NP compression, as discussed above. The decline of $L'_\mathrm{spin} / L$ towards high velocities is more pronounced for large impact parameters, as here the collision is entirely in the ‘shaving’ mode, Fig. 3.

**Figure 9:**
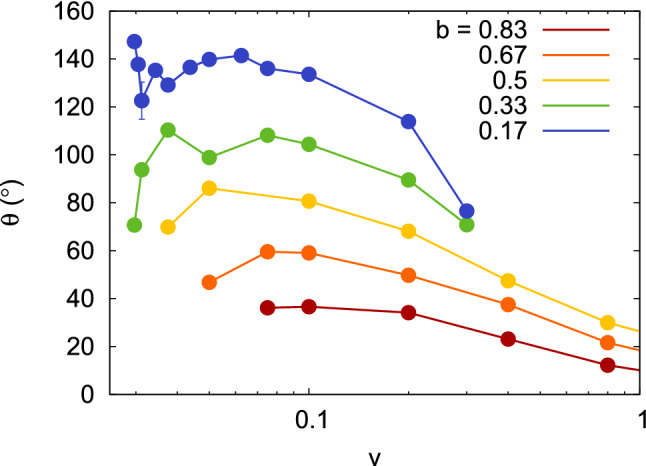
Deflection angle, $\theta$, as a function of collision velocity *v* for several impact parameters *b*. Data are only shown for bouncing collision. Note the logarithmic abscissa scale.

### NP deflection

Due to the collision, the NPs are deflected from their initial direction of motion. The deflection is quantified by the deflection angle $\theta$ defined by

14 $$\begin{aligned} \cos \theta = \frac{\varvec{v} \cdot \varvec{v}'}{v v'} .\end{aligned}$$

The resulting deflection angles are gathered in Fig. 9. They show only few surprises.

1. As for sticking velocities, it is $\varvec{v}'=0$, the deflection angle becomes undefined.
2. For increasing *b*, the angle $\theta$ decreases.
3. For high velocities, the deflection angle tends to zero.

### Bouncing velocity

**Figure 10:**
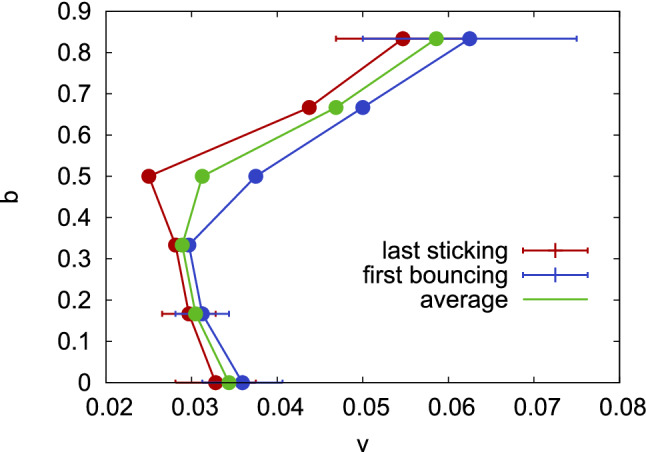
Dependence of largest sticking velocity and smallest bouncing velocity on impact parameters *b*. Their average gives an estimate of the bouncing velocity $v_b$. Error bars for $b=0$, 0.17 and 0.83 are taken from the statistics shown in Fig. 11. This figure provides detailed information on the boundary between sticking and bouncing collisions appearing on the low-velocity side of Fig. 3.

Low-velocity collisions are sticking for all impact parameters, but after passing a critical velocity, $v_b$, the two NPs bounce off each other. At higher velocities, $v \gg v_b$, a second sticking (or fusion) regime shows up for small impact parameters $b \le 0.33$. We will be interested in the (lower) bouncing velocity, since in many cases, the onset of NP bouncing at small velocities is relevant for discussing agglomeration processes^6,15^. In Fig. 10, the highest velocity is marked at which NPs stick and the lowest velocity at which they bounce; we estimate the bouncing velocity as the average between these two.

Often, the bouncing velocity is only discussed for central collisions. There, the discussion proceeds by referring to the Johnson-Kendall-Roberts (JKR) model^47^. It predicts the bouncing velocity of two identical NPs of radius *R* to be determined by the surface energy $\gamma$, the indentation modulus $E_{\mathrm{ind}}$, and mass density $\rho$ via^17,48,49^

15 $$\begin{aligned} v_{b}= \frac{1}{2} \left( \frac{C}{\rho } \right) ^{1/2} \left( \frac{\gamma ^5}{E_{\mathrm{ind}}^2 R^5} \right) ^{1/6} . \end{aligned}$$

The factor of 1/2 enters since velocities in our study correspond to single-particle velocities, while the JKR formula is usually used for relative velocities. (Our previous papers^19–22,35,50,51^ contain an erroneous factor of 2 in the the bouncing velocity, since the velocity in the simulation referred to the single-particle velocity, while the analysis used relative velocities. Thus the bouncing velocities are higher by a factor of 2 than reported in the papers.) Here, *C* is a constant which – depending on the model assumptions – assumes values between 0.30 and 18.3^15,17,48,49^. As we noted previously, we do not find bouncing for central collisions of amorphous LJ spheres of radius $R=88.23$ with unmodified potential; this is in contrast to crystalline LJ NPs^35^. Only by reducing the attractive interaction between the spheres by a factor of 5, cf. Sect. “Method”, we obtained bouncing trajectories. We note that the parameter $\epsilon _{12}$ describing the interparticle interaction is linearly related to the NP surface energy, $\gamma$,^52^, since the surface energy is described by interparticle interactions, $\epsilon _{12}$, rather than the bulk interaction $\epsilon$. We can therefore conclude that it is the high surface energy of NPs that prevents bouncing. Crystalline NPs may bounce, since they show less internal energy dissipation during the collision, such that enough kinetic energy is available for making them bounce.

For amorphous LJ material, it is $\gamma = 1.63$, $E_{\mathrm{ind}}= 53.5$, and $\rho =1.00$^52^; with the reduced interparticle interaction, $\epsilon _{12}=0.2$, the surface energy is reduced to $\gamma = 0.33$. Eq. (15) thus predicts $v_b = 1.39 \cdot 10^{-3} \sqrt{C}$; with the range of *C* quoted above, this gives a range of $v_b$ extending between $0.7 \cdot 10^{-3}$ and $5.4 \cdot 10^{-3}$. Our simulations give a considerably higher value, $v_b=(34 \pm 7) \cdot 10^{-3}$; as mentioned above, amorphous LJ NPs of the size studied here do not bounce at all without reducing the interparticle attraction, and even with reduced attraction, they only bounce at comparatively high velocities. We presume that this is caused by the strong energy dissipation during the collision.

**Figure 11:**
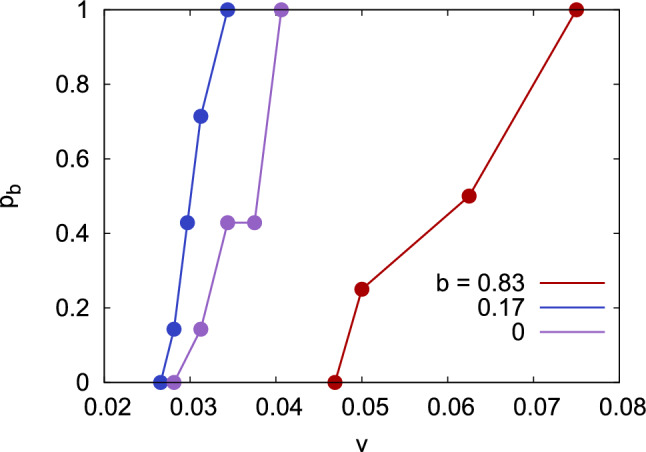
Probability of bouncing, $p_b$, as a function of collision velocity *v* for impact parameters $b=0$, 0.17 and 0.83. The data are based on 7 collisions for $b=0$ and 0.17 and 4 collisions for $b=0.83$.

Interestingly, the bouncing velocity shows a nonmonotonic dependence on impact parameter, see Fig. 10. In order to enhance the statistical reliability of our data, we performed further simulations (up to 6) by rotating both NPs by random angles before colliding them. Figure 11 shows that the bouncing probability $p_b$ – i.e., the fraction of collisions at the same velocity that leads to NP bouncing – increases within a finite velocity range from 0 to 1; in this range, the probability of bouncing depends on the atomistic details of the surface structure of the amorphous NPs. The width of this zone amounts to 0.013 (0.008, 0.028) velocity units for $b=0$ (0.17, 0.83). The data in Fig. 11 corroborate our finding that the bouncing velocity first decreases slightly increasing impact parameter, but increases for grazing collisions.

Previous studies of the impact parameter dependence of the bouncing velocity seem to be rare. Kalweit and Drikakis^18^ found a decrease of $v_b$ with *b* for collisions of crystalline LJ NPs for considerably smaller NP sizes, $R=13.5$. They used an unmodified LJ interaction potential for the interparticle interaction and found bouncing velocities that were considerably higher, ranging between $v=5$ for $b=0.2$ and $v=0.5$ at $b=0.9$. These high values cannot be accounted for by Eq. (15), such that other bouncing mechanisms may prevail in their study as compared to ours.

Kalweit and Drikakis^18^ discuss their results by referring to sticking and bouncing of liquid clusters, for which an extensive body of research exists based on experiments and continuum theories^53–57^. This may be justified since at the high bouncing velocities found in their study^18^, considerable collision-induced heating is reported and also the snapshots shown display apparently molten clusters. For liquid clusters, experimental studies find for central collisions sticking (‘coalescence’)^53,54^; only at higher velocities, bouncing – denoted as ‘reflexive separation’ – occurs^55–57^. For oblique collisions, bouncing may occur, termed ‘stretching separation’. The bouncing velocity obeys a law $v_b \propto 1/b$ based on the idea that ‘the rotational energy must exceed the additional surface energy required to form the initial drops form the coalesced drop pair’^53,58^. This decrease of the bouncing velocity for oblique impact parameters may explain the initial decrease seen in our data, Fig. 10, up to $b \le 0.33$. The increase of $v_b$ towards grazing collisions, $b > 0.5$ must be based on the fact that our NPs are solid and do not melt under the collision; thus we observe NP deformation under the collision which changes the energy balance of rotational and surface energy considered for liquid clusters^53,58^.

## Summary

We studied the collision behavior of amorphous LJ NPs. Since LJ NPs always stick for central impacts, we decreased the interparticle attraction between atoms of differing NPs. We investigated in detail the collisions of two identical NPs with radius $R=88.23$ nm and found the following features.

1. For central impacts, there exists a ‘bouncing window’ ($0.03 \le v \lesssim 0.3$): For too small velocities, NPs stick since their kinetic energy after the collision is too small to surpass the attractive forces; for too large velocity, strong NP deformation dissipates the kinetic energy and the NPs cannot separate after collision. This behavior is similar to that found previously for crystalline LJ NPs^35^.
2. This bouncing window exists up to relative impact parameters of 0.33. For larger impact parameters, high-velocity collisions are always bouncing.
3. The lower bouncing velocity $v_b$ is of particular importance for particle agglomeration processes. It has a non-monotonic dependence on the impact parameter, but changes by less than a factor of 2 from the bouncing velocity at central impact.
4. Due to the amorphous surface structure of the NPs, the bouncing velocity shows some spread, in which the probability of bouncing increases from 0 to 100 %. This spread increases for large impact parameters.
5. Oblique collisions of non-rotating NPs convert angular momentum into NP spin. For low velocities, the NP spin is well described by assuming the NPs to come to a complete stop at the contact point (‘grip’), such that orbital and spin angular momentum share the pre-collision angular momentum in a ratio of 5:2.
6. While central collisions are fully described by the (normal) coefficient of restitution, $e_n$, oblique collisions also require a tangential coefficient of restitution, $e_t$. The latter is fixed in the ‘grip’ regime to a value of 5/7, but increases towards 1 for high-velocity collisions at not too small impact parameters, where the two NPs slide along each other.
7. The normal coefficient of restitution assumes low values in the bouncing window existing for small impact parameters, indicating strong dissipation processes acting in this low-velocity regime.
8. For larger impact parameter and larger velocities, the normal coefficient of restitution changes sign, since the two NPs do not repel but their motion direction persists.

These results may serve to outline the generic behavior of collisions between amorphous nanoparticles. The collision dynamics of amorphous NPs may be easier to study than that of crystalline particles, since collision results will in general depend on the crystalline faces with which the 2 NPs encounter each other^35^. From a computational point of view, the NPs employed here are quite large, containing more than 3 million atoms each. The results depart in detail from previous results of NPs containing only $10^4$ atoms^18^; there collisional heating rendered the NPs liquid during the collision, affecting the dynamics in particular at large-velocity collisions. But also the low-velocity bouncing velocity differed from the present results obtained for larger NPs in that it showed a monotonic decrease with increasing impact parameter.

In future work, it will be interesting to compare the present generic results with simulations for concrete materials, such as silica or water ice. Also, it might be relevant to extend this study to liquid NPs, in order to make contact with available data and theories of collisions between liquid droplets.

## Data Availability

All data used for this study are contained in this article.

## References

[1] O’Dowd Colin D, Wagner Paul E (2007). Nucleation and atmospheric aerosols.

[2] Lazaridis Mihalis, Drossinos Yannis (2014). Aerosol dynamics, in Aerosol Science, edited by Ian Colbeck and Mihalis Lazaridis (John Wiley). Chap..

[3] Ko Higashitani, Hisao Makino, and Shuji Matsusaka, eds., Powder Technology Handbook (CRC Press, 2019).

[4] Philip J (2010). Armitage, Astrophysics of planet formation.

[5] Armitage Philip J (2011). Dynamics of protoplanetary disks. Annu. Rev. Astron. Astrophys..

[6] Blum Jürgen (2010). Dust growth in protoplanetary disks - a comprehensive experimental / theoretical approach. Res. Astron. Astrophys..

[7] Birnstiel T, Fang M, Johansen A (2016). Dust evolution and the formation of planetesimals. Space Sci. Rev..

[8] Nakamura Eizo, Makishima Akio, Moriguti Takuya, Kobayashi Katsura, Tanaka Ryoji, Kunihiro Tak, Tsujimori Tatsuki, Sakaguchi Chie, Kitagawa Hiroshi, Ota Tsutomu, Yachi Yusuke, Yada Toru, Abe Masanao, Fujimura Akio, Ueno Munetaka, Mukai Toshifumi, Yoshikawa Makoto, Kawaguchi Jun’ichiro (2012). Space environment of an asteroid preserved on micrograins returned by the Hayabusa spacecraft. Procee. Natl. Acad. Sci..

[9] Gáspár A, Rieke GH, Balog Z (2013). The collisional evolution of debris disks. Astrophys. J..

[10] Larry W (2010). Esposito, “Composition, structure, dynamics, and evolution of Saturn’s rings”. Annu. Rev. Earth Planet. Sci..

[11] Bentley Mark S, Schmied Roland, Mannel Thurid, Torkar Klaus, Jeszenszky Harald, Romstedt Jens, Levasseur-Regourd Anny- Chantal, Weber Iris, Jessberger Elmar K, Ehrenfreund Pascale, Koeberl Christian, Havnes Ove (2016). Aggregate dust particles at comet 67P/Churyumov-Gerasimenko. Nature.

[12] Langevin Y, Hilchenbach M, Ligier N, Merouane S, Hornung K, Engrand C, Schulz R, Kissel J, Rynö J, Eng P (2016). Typology of dust particles collected by the COSIMA mass spectrometer in the inner coma of 67P/Churyumov Gerasimenko. Icarus.

[13] Poppe Torsten, Blum Jürgen, Henning Thomas (2000). Analogous experiments on the stickiness of micron-sized preplanetary dust. Astrophys. J..

[14] Beitz E, Güttler C, Blum J, Meisner T, Teiser J, Wurm G (2011). Low-velocity collisions of centimeter-sized dust aggregates. Astrophys. J..

[15] Dominik C, Tielens AGGM (1997). The physics of dust coagulation and the structure of dust aggregates in space. Astrophys. J..

[16] Schwager Thomas, Pöschel Thorsten (2008). Coefficient of restitution for viscoelastic spheres: The effect of delayed recovery. Phys. Rev. E.

[17] Krijt S, Güttler C, Heißelmann D, Dominik C, Tielens AGGM (2013). Energy dissipation in head-on collisions of spheres. J. Phys. D.

[18] Kalweit Marco, Drikakis Dimitris (2006). Collision dynamics of nanoscale Lennard-Jones clusters. Phys. Rev. B.

[19] Nietiadi Maureen L, Umstätter Philipp, Tjong Tiffany, Rosandi Yudi, Millán Emmanuel N, Bringa Eduardo M, Urbassek Herbert M (2017). The bouncing threshold in silica nanograin collisions. Phys. Chem. Chem. Phys..

[20] Nietiadi Maureen L, Umstätter Philipp, Alhafez Iyad Alabd, Rosandi Yudi, Bringa Eduardo M, Urbassek Herbert M (2017). Collision-induced melting in collisions of water ice nanograins: Strong deformations and prevention of bouncing. Geophys. Res. Lett..

[21] Nietiadi Maureen L, Rosandi Yudi, Urbassek Herbert M (2020). Collisions between ice-covered silica grains: An atomistic study. Icarus.

[22] Nietiadi Maureen L, Rosandi Yudi, Bringa Eduardo M, Urbassek Herbert M (2022). Peripheral collisions of icecovered silica dust grains. Astrophys. J..

[23] C. Güttler, D. Heißelmann, J. Blum, and S. Krijt, “Normal collisions of spheres: A literature survey on available experiments,” arXiv:1204.0001 (2013).

[24] Frenkel D, Smit B (2002). Understanding molecular simulation.

[25] Mastny Ethan A, Juan J, de Pablo Juan J (2007). Melting line of the Lennard-Jones system, infinite size, and full potential. J. Chem. Phys..

[26] Wang Xipeng, Ramirez-Hinestrosa Simon, Dobnikar Jure, Frenkel Daan (2020). The Lennard-Jones potential: when (not) to use it. Phys. Chem. Chem. Phys..

[27] Yi Min-Young, Kim Dong-Sik, Lee Jin-Won, Koplik Joel (2005). Molecular dynamics (MD) simulation on the collision of a nano-sized particle onto another nano-sized particle adhered on a flat substrate. Aerosol Sci..

[28] Kuninaka Hiroto, Hayakawa Hisao (2009). Simulation of cohesive head-on collisions of thermally activated nanoclusters. Phys. Rev. E.

[29] Saitoh Kuniyasu, Bodrova Anna, Hayakawa Hisao, Brilliantov Nikolai V (2010). Negative normal restitution coefficient found in simulation of nanocluster collisions. Phys. Rev. Lett..

[30] Tanaka H, Wada K, Suyama T, Skuzumi O (2012). Growth of cosmic dust aggregates and reexamination of particle interaction models. Prog. Theor. Phys. Suppl..

[31] Jung Seung-Chai, Bang Jong-Geun, Yoon Woong-sup (2012). Applicability of the macro-scale elastic contact theories for the prediction of nano-scaled particle collision with a rigid flat surface under non-adhesive and weakly-adhesive conditions. J. Aerosol Sci..

[32] Takato Yoichi, Sen Surajit, Lechman Jeremy B (2014). Strong plastic deformation and softening of fast colliding nanoparticles. Phys. Rev. E.

[33] Takato Yoichi, Benson Michael E, Sen Surajit (2015). Rich collision dynamics of soft and sticky crystalline nanoparticles: Numerical experiments. Phys. Rev. E.

[34] Takato Yoichi, Benson Michael E, Sen Surajit (2018). Small nanoparticles, surface geometry and contact forces. Proc. R. Soc. A.

[35] Nietiadi Maureen L, Millán Emmanuel N, Bringa Eduardo M, Urbassek Herbert M (2019). Bouncing window for colliding nanoparticles: Role of dislocation generation. Phys. Rev. E.

[36] Halicioglu T, Pound GM (1975). Calculation of potential energy parameters from crystalline state properties. Phys. stat. sol. (a).

[37] Guan P, Mckenzie DR, Pailthorpe BA (1996). MD simulations of Ag film growth using the Lennard-Jones potential. J. Phys.: Condens. Matter..

[38] Millán Emmanuel N, Tramontina Diego R, Urbassek Herbert M, Bringa Eduardo M (2016). The elastic-plastic transition in nanoparticle collisions. Phys. Chem. Chem. Phys..

[39] Urbassek HM, Waldeer KT (1991). Spikes in condensed rare gases induced by keV-atom bombardment. Phys. Rev. Lett..

[40] Anders C, Urbassek HM, Johnson RE (2004). Linearity and additivity in cluster-induced sputtering: A molecular-dynamics study of van der Waals bonded systems. Phys. Rev. B.

[41] St. Plimpton, Fast parallel algorithms for short-range molecular dynamics. J. Comput. Phys. 117, 1-19 (1995), http://lammps.sandia.gov/ .

[42] A. Stukowski, Visualization and analysis of atomistic simulation data with OVITO - the Open Visualization Tool. Model. Simul. Mater. Sci. Eng. 18, 015012 (2010), http://www.ovito.org/ .

[43] Johnson KL (1985). Contact mechanics.

[44] Foerster Samuel F, Louge Michel Y, Chang Hongder, Allia Khédidja (1994). Measurements of the collision properties of small spheres. Phys. Fluids.

[45] Cross Rod (2019). Oblique impact of a steel ball. Powder Technol..

[46] Cross Rod (2022). Two-dimensional collisions of disks and spheres. Eur. J. Phys..

[47] Johnson KL, Kendall K, Roberts AD (1971). Surface energy and the contact of elastic solids. Proc. R. Soc. London. Ser. A.

[48] Thornton C, Ning Z (1998). A theoretical model for the stick/bounce behaviour of adhesive, elastic-plastic spheres. Powder Technol..

[49] Brilliantov Nikolai V, Albers Nicole, Spahn Frank, Pöschel Thorsten (2007). Collision dynamics of granular particles with adhesion. Phys. Rev. E.

[50] Nietiadi Maureen L, Valencia Felipe, Gonzalez Rafael I, Bringa Eduardo M, Urbassek Herbert M (2020). Collisions between amorphous carbon nanoparticles: phase transformations. A &A.

[51] Nietiadi Maureen L, Rosandi Yudi, Urbassek Herbert M (2020). Bouncing of hydroxylated silica nanoparticles: an atomistic study based on REAX potentials. Nanoscale Res. Lett..

[52] Umstätter Philipp, Urbassek Herbert M (2021). Molecular dynamics of rolling and twisting motion of amorphous nanoparticles. Sci. Rep..

[53] P. R. Brazier-Smith, S. G. Jennings, and J. Latham, The interaction of falling water drops: coalescence. Proceedings of the Royal Society of London. A. Mathematical and Physical Sciences 326, 393-408 (1972).

[54] Jiang YJ, Umemura A, Law CK (1992). An experimental investigation on the collision behaviour of hydrocarbon droplets. J. Fluid Mech..

[55] Ashgriz N, Poo JY (1990). Coalescence and separation in binary collisions of liquid drops. J. Fluid Mech..

[56] Estrade JP, Carentz H, Lavergne G, Biscos Y (1999). Experimental investigation of dynamic binary collision of ethanol droplets - a model for droplet coalescence and bouncing. Int. J. Heat Fluid Flow.

[57] Sommerfeld Martin, Kuschel Matthias (2016). Modelling droplet collision outcomes for different substances and viscosities. Exp. Fluids.

[58] Suo Shaoyi, Jia Ming (2020). Correction and improvement of a widely used droplet-droplet collision outcome model. Phys. Fluids.

